# The regulation of muscle mass by endogenous glucocorticoids

**DOI:** 10.3389/fphys.2015.00012

**Published:** 2015-02-03

**Authors:** Theodore P. Braun, Daniel L. Marks

**Affiliations:** ^1^Department of Internal Medicine, University of Washington Medical CenterSeattle, WA, USA; ^2^Papé Family Pediatric Research Institute, Oregon Health and Science UniversityPortland, OR, USA

**Keywords:** glucococorticoids, muscle, skeletal, HPA-axis, cachexia, catabolism, wasting, protein synthesis

## Abstract

Glucocorticoids are highly conserved fundamental regulators of energy homeostasis. In response to stress in the form of perceived danger or acute inflammation, glucocorticoids are released from the adrenal gland, rapidly mobilizing energy from carbohydrate, fat and protein stores. In the case of inflammation, mobilized protein is critical for the rapid synthesis of acute phase reactants and an efficient immune response to infection. While adaptive in response to infection, chronic mobilization can lead to a profound depletion of energy stores. Skeletal muscle represents the major body store of protein, and can become substantially atrophied under conditions of chronic inflammation. Glucocorticoids elicit the atrophy of muscle by increasing the rate of protein degradation by the ubiquitin-proteasome system and autophagy lysosome system. Protein synthesis is also suppressed at the level of translational initiation, preventing the production of new myofibrillar protein. Glucocorticoids also antagonize the action of anabolic regulators such as insulin further exacerbating the loss of protein and muscle mass. The loss of muscle mass in the context of chronic disease is a key feature of cachexia and contributes substantially to morbidity and mortality. A growing body of evidence demonstrates that glucocorticoid signaling is a common mediator of wasting, irrespective of the underlying initiator or disease state. This review will highlight fundamental mechanisms of glucocorticoid signaling and detail the mechanisms of glucocorticoid-induced muscle atrophy. Additionally, the evidence for glucocorticoids as a driver of muscle wasting in numerous disease states will be discussed. Given the burden of wasting diseases and the nodal nature of glucocorticoid signaling, effective anti-glucocorticoid therapy would be a valuable clinical tool. Therefore, the progress and potential pitfalls in the development of glucocorticoid antagonists for muscle wasting will be discussed.

## Introduction

Energy balance in vertebrate animals is a highly regulated process that is differentially modulated to respond to a diverse array of challenges. Fundamentally, organisms must risk their current energy stores to find food, avoid predation, fight infection, and to reproduce. Given the critical importance of energy metabolism to organismal fitness, numerous evolutionary pressures have acted on this process. One of the most profound challenges to mammalian energy homeostasis is acute infection. A multitude of behavioral and metabolic changes occur that greatly tax energy reserves. The febrile response is a fundamental mediator of innate immunity. It is estimated that a 13% increase in metabolic rate is required for every 1° increase in body temperature (Kluger, 1978). Simultaneously, the immune system must become anabolic, producing granulocytes, antibodies and acute phase proteins to combat the infection. This dramatic increase in energy consumption comes in the face of the seemingly paradoxical behavioral response of a decrease in appetite (Hart, 1988). There are a number of possible purposes to this behavior, including a decreased risk of predation during this period and the energetic cost of finding and digesting food (Exton, 1997). Regardless of the evolved purpose, this leads to a marked imbalance between energy intake and expenditure draws heavily on energy stores, and in particular skeletal muscle (Baracos et al., 1983).

**Figure 1:**
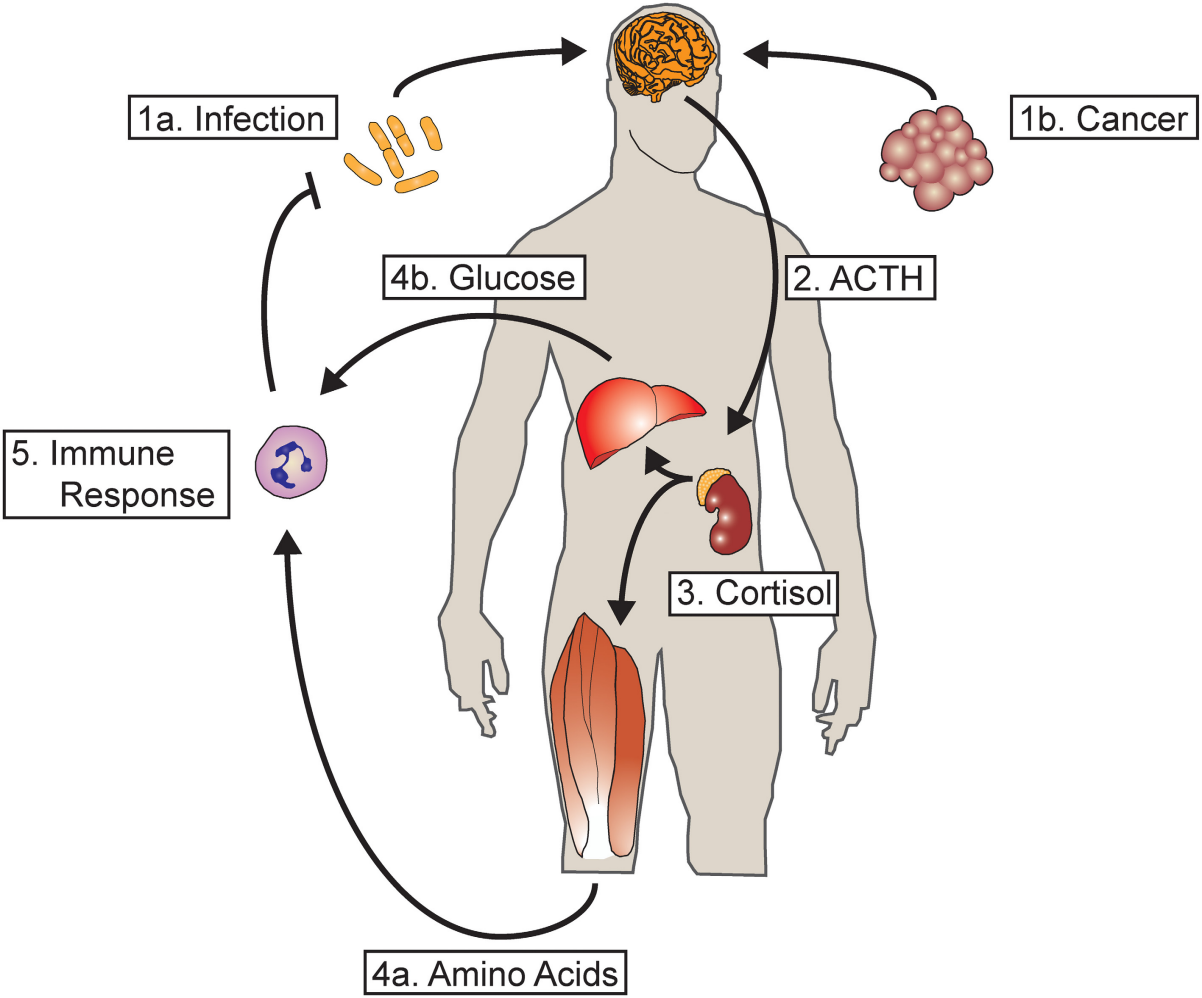
**Glucocorticoids mediate a metabolic response to acute and chronic inflammation. (1a)** During acute infection, inflammatory mediators act in the central nervous system activating hypothalamic CRH neurons. **(1b)** Chronic inflammation, such as from tumor growth, also activates hypothalamic CRH neurons. **(2)** The release of CRH into the pituitary portal vasculature results in the release of ACTH by the anterior pituitary into the systemic circulation. **(3)** ACTH acts on the adrenal gland, resulting in the release of cortisol (or corticosterone in rodents). **(4a)** Cortisol acts on skeletal muscle, resulting in the breakdown of contractile protein and the mobilization of amino acids. **(4b)** Cortisol also acts on the liver to increase hepatic gluconeogenesis. **(5)** The resultant substrate mobilized from energy reserves serves to fuel the synthesis of acute phase reactants as well as the innate and adaptive immune response. In the case of acute infection, the resultant feedback loop results in resolution and clearance of the offending organism, terminating the inflammatory response and the mobilization of resources. However, in cases of chronic inflammation such as cancer where the immune response is unable to terminate the underlying inflammatory stimulus, continuous mobilization of amino acids leads to a profound depletion of skeletal muscle.

Endogenous glucocorticoids are one of the most primitive regulators of energy homeostasis. Named for their ability to increase serum glucose, glucocorticoids have important roles in modifying carbohydrate, fat and protein metabolism as well as numerous other physiologic functions. Glucocorticoid receptors (GR) are conserved down to teleosts where they play a similar role in metabolism (Wendelaar Bonga, 1997). Cortisol, the physiologic glucocorticoid in humans, is produced by the zona fasiculata of the adrenal cortex in response to adrenocorticotrophic hormone (ACTH) released by the anterior pituitary. ACTH, in turn is regulated by the hypothalamic production and release of corticotropin-releasing hormone forming the apical component of the hypothalamic-pituitary-adrenal axis (HPA axis). Numerous stressors activate the HPA axis, releasing cortisol and mobilizing energy reserves. Skeletal muscle serves as a major body store of amino acids, and glucocorticoids act to induce the catabolism of this tissue, increasing the plasma levels of free amino acids (Wise et al., 1973). Under conditions of starvation, HPA activation and subsequent proteolysis in skeletal muscle provides amino acid substrate for hepatic gluconenogenesis (Pilkis et al., 1988). The HPA axis is also activated in response to inflammation, with the released amino acids fueling the synthesis of acute phase reactants (Figure 1) (Besedovsky et al., 1986).

While adaptive in the setting of starvation or acute infection, prolonged mobilization of muscle protein resulting from chronic infection or glucocorticoid pharmacotherapy results in a profound loss of muscle mass, functional impairment and mortality (Figure 1). A defining feature of the cachexia syndrome, this condition complicates, the vast majority of chronic diseases. In addition to decreased quality of life, the presence of muscle atrophy portends a negative outcome in numerous chronic diseases (Tan et al., 2009; Noori et al., 2010; Fülster et al., 2013). It is important to distinguish muscle atrophy from inflammatory myopathy. The former involves a reduction in the size of muscle fibers without disruption of the cell membrane, while in the latter there is a marked immune cell infiltration into muscle and loss of membrane integrity. This review will focus on the contribution of glucocorticoid signaling to the pathogenesis of muscle atrophy in chronic disease. Given the paucity of treatments for cachexia, we will highlight current avenues of research with the potential to offer therapeutic interventions in the future.

## Molecular mechanisms of glucocorticoid-induced atrophy

### Inflammatory regulation of the HPA axis

Activation of the HPA axis by inflammation is a well-described phenomenon with an extensive history of work. Through a series of elegant studies, it was demonstrated that the prototypical cytokine interleukin-1β activates the HPA axis via the regulation of CRH release without clear evidence for activity at the level of the pituitary. The release of ACTH by pituitary and subsequent release of corticosterone (the predominant glucocorticoid in rodents) by the adrenal gland is blocked by immunoneutralization of CRH (Berkenbosch et al., 1987; Sapolsky et al., 1987). Cytokines generated in the periphery likely activate CRH neurons in a number of ways. A particularly well-characterized mechanism involves the induction of cyclooxygenase in the cerebrovascular endothelium of the brain stem, activating ascending projections that converge on CRH neurons (Ericsson et al., 1995, 1997; García-Bueno et al., 2009). Genetic inhibition of cytokine signaling in endothelium prevents the activation of the HPA-axis by inflammatory cytokines (Ching et al., 2007). Cytokines can also gain access to the CNS via areas of permeable blood brain barrier known as circumventricular organs, and act upon local neuronal populations that project to hypothalamic CRH neurons (Buller, 2001). Finally, peripheral inflammation can directly activate afferent projections originating in the periphery, thereby modulating HPA axis activation. The vagus nerve expresses toll-like receptor 4 (TLR4), the cellular receptor for endotoxin, and vagotomy eliminates some of the behavioral response to peripheral inflammation in certain models (Watkins et al., 1995). Other studies have demonstrated that certain cytokines such as interleukin-6 and leukemia inhibitory factor also exert a direct effect on the pituitary (Akita et al., 1995; Bethin et al., 2000). Thus, a series of mechanisms that are differentially evoked (based on the nature and site of the inflammatory challenge), converge on the HPA axis, resulting in the release of glucocorticoids.

### Glucocorticoids as mediators of atrophy

Glucucocorticoid-induced muscle atrophy was first noted in the setting of Cushing's Disease, were pituitary adenomas overproduce ACTH (Cushing, 1994). It was not until the 1950's that metabolic tracing studies demonstrated the ability exogenously administered glucocorticoids and ACTH to promote protein degradation in muscle while simultaneously preventing protein synthesis (Hoberman, 1950). Simultaneously, with increasing pharmacologic use of glucocorticoids, came the recognition of muscle atrophy as a side effect of glucocorticoid treatment in the clinical entity steroid myopathy. However, the first evidence that endogenous glucocorticoids contributed to the process of muscle atrophy came in the observation that adrenalectomized animals failed to undergo muscle atrophy in response to fasting (Wing and Goldberg, 1993). Subsequent studies demonstrated an integral role for endogenous glucocorticoids as fundamental mediators of muscle atrophy in multiple conditions, as covered in detail in a following section.

### Tissue specific modulation of glucocorticoid activity by hydroxysteroid dehydrogenase enzymes

The activity of glucocorticoids in tissues is modulated by two enzymatic activities that interconvert cortisol (corticosterone in rodents) with its inactive metabolite cortisone (11-dehydro-corticosterone in rodents). The enzyme 11-beta hydroxysteroid dehydrogenase 1 (11β-HSD1) converts cortisone (that does not bind the GR) to cortisol by hydrogenating the carbonyl oxygen at the 11 position. It is expressed in numerous metabolic tissues including liver, adipose tissue and brain. Additionally, it is expressed in skeletal muscle where it plays an integral role in the regulation of muscle atrophy. Cortisone is capable of inducing proteolysis and atrophy in cultured myotubes, an effect that is abolished in the presence of an inhibitor of 11β-HSD1 (Biedasek et al., 2011). Skeletal muscle 11β-HSD1 also modulates glucocorticoid-induced insulin resistance, which as discussed below, likely impacts atrophy (Morgan et al., 2009). 11β-HSD2 converts active cortisol into inactive cortisone, decreasing the quantity of active glucocorticoid reaching the receptor. The best-described physiologic role for 11β-HSD2 is in tissues responding to the mineralocorticoid aldosterone. Cortisol, despite being the predominant circulating glucocorticoid in humans, binds to the mineralocorticoid receptor with higher affinity than it does to the GR. Therefore, in tissues such as the renal collecting duct, 11β-HSD2 inactivates cortisol allowing mineralocorticoid receptor signaling to be governed by circulating aldosterone concentrations (Chapman et al., 2013). The role (or indeed whether it is expressed at all) of 11β-HSD2 in skeletal muscle is not well described, and represents an exciting potential avenue for investigation.

### Fiber-type specificity of glucocorticoid-induced muscle atrophy

Glucocorticoids have a particular predilection for inducing the atrophy of fast-twitch skeletal muscle, with slow twitch muscle and to a greater extent cardiac muscle being largely protected (Sandri et al., 2006; Shimizu et al., 2011). There is some teleologic sense to this specificity, as fast twitch muscle may be more expendable given the role of slow twitch muscle in maintenance of posture and respiration. An apparent explanation for this discordance in atrophy comes from the differential expression of the GR between fiber types. While fast twitch muscles express abundant GR, expression is relatively low in slow twitch muscles (Sandri et al., 2006; Shimizu et al., 2011). An alternate explanation arises from the expression of the transcriptional co-activator PGC-1α in slow but not fast twitch fibers. Forced expression of PGC-1α protects against fasting-induced atrophy by suppressing the transcriptional activation of the atrophy program which is discussed in detail below (Sandri et al., 2006).

### Direct transactivation of catabolic regulators

Within the context of the muscle cell, glucocorticoids induce muscle atrophy through a variety of mechanisms. The GR is a type I nuclear hormone receptor, and as such is localized to the cytosol in the absence of ligand. However, upon ligand binding, it translocates to the nucleus where interacts with specific DNA sequences known as glucocorticoid response elements (GREs) (recently reviewed in Kadmiel and Cidlowski, 2013). Once bound, it assembles a complex of coactivators and mediators ultimately resulting in the recruitment of RNA polymerase II and the initiation of transcription. An equally important mechanism by which the ligand bound GR mediates gene transcription is via the repression of other transcription factor complexes which occurs independent of GR DNA binding. The best studied of these are NF-κB and AP-1 which regulate an overlapping set of genes governing inflammation, growth and proliferation. Ligand bound GR interferes with the binding of the p65 subunit of NF-κB to the basal transcriptional machinery, preventing it from activating target inflammatory genes (De Bosscher et al., 2000). Similarly, the GR interacts with c-Jun a component of the AP-1 complex, preventing it from driving transcription of target genes (Wei et al., 1998). Classically it has been believed that the metabolic effects of glucocorticoids are dependent on DNA binding and transcriptional activation, while the anti-inflammatory effects depend on DNA binding-independent repression. However, as is discussed in detail below, recent work has called this mechanistic separation into question.

### Muscle-specific ubiquitin ligases regulate atrophy

**Figure 2:**
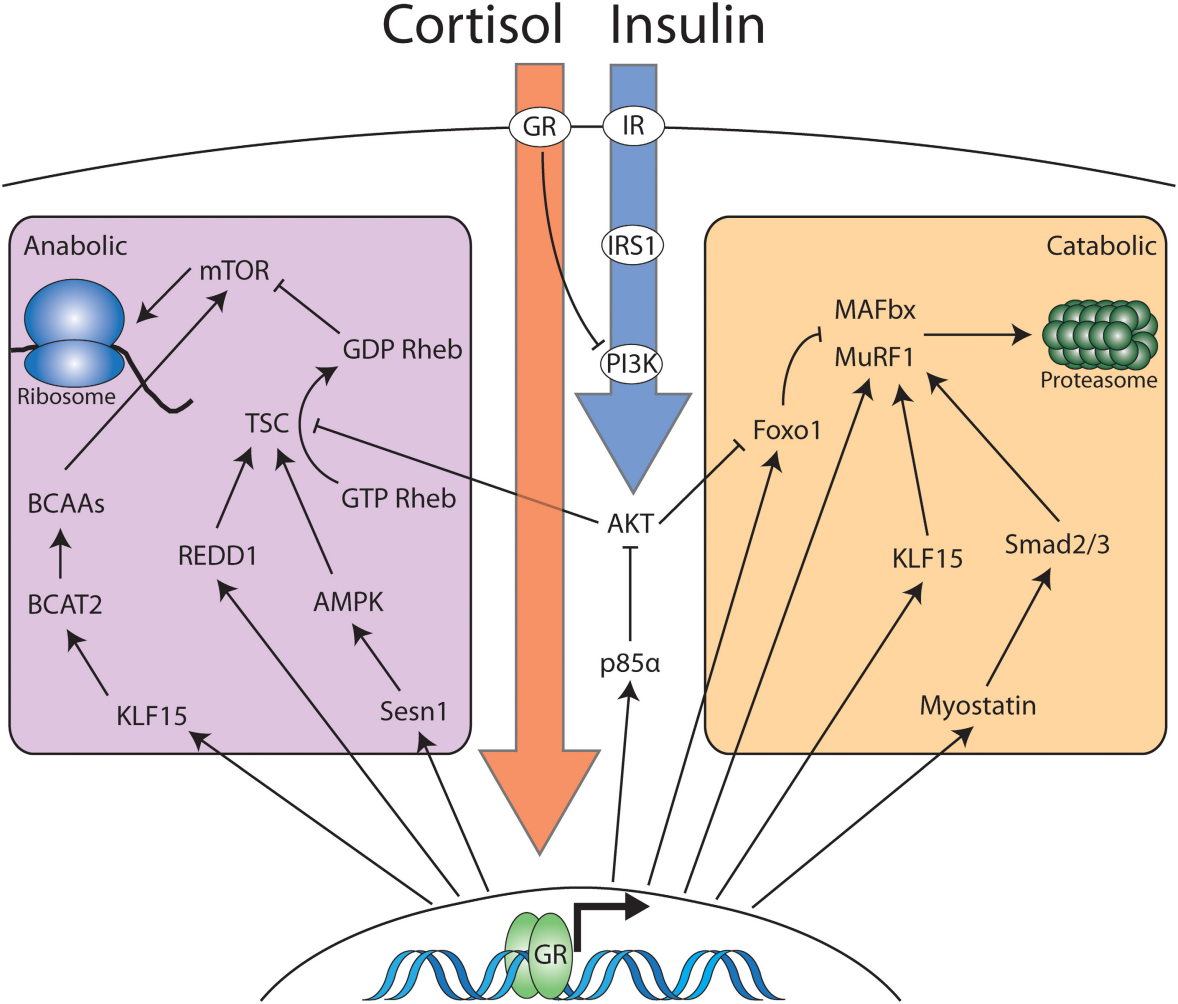
**Cortisol and insulin signaling interact to modulate the anabolic and catabolic balance in skeletal muscle**. Cortisol and corticosterone interact with cytosolic glucocorticoid receptor in skeletal muscle. The majority of the effects of cortisol occur via glucocorticoid receptor dimerization, nuclear localization and activation of transcription. However, cytosolic ligand bound GR monomers also antagonize insulin signaling at the level of PI3K. Nuclear GR induces the transcription of multiple genes that partition into two distinct domains: Genes that increase catabolic processes and genes that decrease anabolic processes. Increased anabolic activity occurs via increased ubiquitin-proteasome activity or increases in the activity of the autophagy-lysosome system (not pictured). The reduction in anabolic activity occurs via a number of pathways that converge to inhibit mTOR-dependent protein translation. Insulin signaling opposes glucocorticoid dependent mobilization of muscle protein via interaction with membrane bound insulin receptor. Signaling downstream of the insulin receptor results in inhibition of proteasomal targeting as well as stimulating mTOR-dependent protein translation.

Early research into the molecular mechanisms of muscle atrophy focused on protein degradation, and which occurs largely as the result of activation of the ubiquitin proteasome system (UPS) (Although there has been renewed interest in the contribution of the autophagy-lysosome system recently, as discussed below). While many components of the UPS including proteasomal subunits are increased in expression under atrophic conditions, the regulators that mediate the specificity of this reaction for myofibrillar protein remained elusive. Subsequent screening efforts lead to the discovery of two E3 ubiquitin ligases Muscle Atrophy F-box (MAFbx/atrogin-1) and Muscle Specific Ring Finger Protein 1 (MuRF1) as being highly up-regulated in muscle undergoing atrophy (Figure 2) (Bodine et al., 2001; Gomes et al., 2001). Both are expressed exclusively in skeletal muscle and requisite for the process of normal atrophy (Jagoe et al., 2002; Lecker et al., 2004; Sacheck et al., 2007). MuRF1 directly participates in the ubiquitination and degradation of heavy chain myosin and numerous heavy chain regulatory proteins (Clarke et al., 2007; Cohen et al., 2009). In response to dexamethasone treatment, MAFbx and MuRF1 play divergent roles. MuRF1 knockout mice are resistant to dexamethasone-induced muscle atrophy with preservation of muscle mass, fiber cross sectional area and contractile properties (Baehr et al., 2011). Interestingly, deletion of MAFbx does not protect against muscle atrophy in response to glucocorticoid administration despite the significant protection against denervation-induced atrophy observed in MAFbx knockout mice (Bodine et al., 2001). Furthermore, a distinct gene expression profile is observed in MuRF1 knockout mice administered dexamethasone, including alterations in genes involved with the neuromuscular junction and fat metabolism (Furlow et al., 2013). This argues that MuRF1 also plays a critical role in regulating gene expression in atrophic muscle.

In addition to regulating protein breakdown, both muscle specific E3 ligases also contribute to the decrease in protein synthesis in response to glucocorticoid administration. Interestingly, the principle role of MAFbx in muscle atrophy may be the regulation of protein synthesis as, no MAFbx targets have been identified that would allow it to directly participate in protein degradation. The only described substrates for this E3 ubiquitin ligase are MyoD and eIF3-f controlling myogenic differentiation and protein translation respectively (Guttridge et al., 2000; Csibi et al., 2009). Using a yeast two-hydrid screen, eIF3-f was identified as a MAFbx interacting protein. In cultured myotubes subjected to glucocorticoid treatment, MAFbx is rapidly upregulated with a concurrent increase in ubiquitinated eIF3-f and a decrease in total eIF3-f protein (Lagirand-Cantaloube et al., 2008). This is dependent on multiple C-terminal lysine residues that serve as the sites for polyubiquitination (Csibi et al., 2009). eIF3-f interacts directly with the 40S ribosome and serves a critical function in the formation of the pre initiation complex. Therefore, the selective degradation of eIF3-f results in a global suppression of translational initiation and subsequent protein synthesis. MuRF1 also mediates the suppression of protein synthesis in response to glucocorticoid administration. MuRF1 knockout mice resist the suppression of protein synthesis that occurs with dexamethasone administration, perhaps via the aforementioned regulation of gene expression in muscle undergoing atrophy (Baehr et al., 2011).

### Transcriptional regulation of muscle specific E3 ligases

The upregulation of MAFbx and MuRF1 in response to glucocorticoid administration occurs as the result of the concerted action of a series of transcription factors. A GRE is present in the promoter region of the MuRF1 gene, and is responsive to glucocorticoid administration (Waddell et al., 2008; Shimizu et al., 2011). However, no GRE has been identified in the MAFbx promoter demonstrating that additional transcription factors are requisite for initiation of the atrophy program. The Forkhead-box (Foxo) family of transcription factors plays a critical role in regulating muscle atrophy (Sandri et al., 2004). The activity of this transcription factor family is regulated via both transcriptional and post-translational mechanisms. Upstream growth factors such as insulin and IGF-1 (detailed below) inhibit Foxo activity via a signal transduction pathway culminating in Foxo phosphorylation and nuclear exclusion. Additionally, Foxo transcription factors are upregulated at the gene expression level in response to glucocorticoid treatment (Lecker et al., 2004; Braun et al., 2011). A putative GRE has been identified in the Foxo3a promoter, although the activity of this site in muscle has yet to be confirmed (Lützner et al., 2012). Transgenic overexpression of Foxo1 in skeletal muscle is sufficient to drive muscle atrophy, arguing that both Foxo phosphorylation and total expression level govern the activity of these transcription factors. Foxo transcription factors increase the transcription of the muscle-specific E3 ligases via binding to conserved elements in the promoter and the assembly of the transcriptional apparatus. The MAFbx promoter contains Foxo binding sites that are required for activation of MAFbx expression (Sandri et al., 2004). The GRE and Foxo binding elements in the MuRF1 promoter are close in proximity and interact synergistically to induce MuRF1 expression in response to glucocorticoid treatment (Waddell et al., 2008).

Glucocorticoids also regulate the expression of MAFbx and MuRF1 via a transcription factor known as Krüppel-like factor 15 (KLF15) (Shimizu et al., 2011). Ligand bound GR induces the transcription of KLF15, which subsequently interacts with the promoter regions of both MAFbx and MURF1 to induce their expression. Furthermore, overexpression of constitutively active forms of both transcription factors results in additive increases in MAFbx and MuRF1 promoter activity and expression. KLF15 overexpression also results in increased expression of Foxo1 and Foxo3a, demonstrating further crosstalk between these transcription factors. It is the synergistic interplay between ligand bound glucocorticoids and this induced transcription factor network that regulates muscle mass via the activation of MAFbx and MuRF1.

It has recently come to light that glucocorticoids may also activate inflammatory signaling pathways as mechanism of inducing atrophy. The adaptor protein TRAF6 is classically thought to couple inflammatory signals from membrane bound cytokine receptors to down-stream transcription factor networks inducing the inflammatory response such as NF-κB. However, pharmacologic doses of dexamethasone increase TRAF6 expression, and TRAF6 knockdown attenuates dexamethasone-induced atrophy both *in vitro* and *in vivo* (Sun et al., 2014). The demonstration that glucocorticoid signaling activates inflammatory pathways argues against the exclusively anti-inflammatory role of glucocorticoids. Further, it brings clarity to previously disparate data demonstrating that both glucocorticoids and NF-κB are necessary and sufficient for inflammatory muscle atrophy by suggesting they act in the same pathway (Cai et al., 2004; Braun and Marks, 2010; Braun et al., 2013).

### Autophagy as a mechanism of protein breakdown in skeletal muscle

Autophagocytic digestion of contractile proteins and organelles also plays a critical role in the process of muscle atrophy, and shares similar regulatory machinery [For a full discussion of autophagy and muscle atrophy, see the following recent review (Sandri, 2010)]. Foxo3 induces the expression of Bnip3, LC3 and Gabarapl1 in skeletal muscle undergoing atrophy, leading to increased autophagosome assembly and lysosomal fusion (Mammucari et al., 2007). Foxo3a directly regulates LC3 gene expression by interacting with Foxo binding elements in its promoter. Interestingly, mitochondrial autophagy (mitophagy) was recently identified as a contributor to muscle atrophy, with the E3 ubiquitin ligase Mul1 playing a fundamental regulatory role (Lokireddy et al., 2012). The treatment of cultured myotubes with dexamethasone induces Mul1 expression and induces mitophagy. Foxo transcription factors also regulate mitophagy via interaction with the Mul1 promoter and regulation of expression. A complete description of the role of autophagy in muscle atrophy is beyond the scope of this review. However, it is clear that autophagy plays an important role in mediating the breakdown of protein in response to glucocorticoid treatment, acting in concert with the ubiquitin proteasome system.

### Glucocorticoid-mediated inhibition of anabolic pathways

Although there was initially more interest in catabolic pathways in skeletal muscle, there has been renewed interest in understanding the suppression of protein synthesis as a mechanism of muscle atrophy. A central player in the regulation of global protein synthesis is the mammalian target of rapamycin (mTOR) complex. The activity of the mTOR is regulated by the TSC1/2 complex, which functions as a GTPase-activating protein (GAP) for Rheb. (The basic mechanisms of mTOR signaling have been recently reviewed in Laplante and Sabatini, 2012). GTP-bound Rheb stimulates mTOR activity, while GDP-Rheb inhibits it. Thus, assembly of the TSC1/2 complex increases GDP-Rheb, inhibiting mTOR activity. Regulated in Development and DNA Damage Responses (REDD1) is significantly upregulated in response to glucocorticoid administration and has been identified as a direct glucocorticoid-target gene by several studies (Shimizu et al., 2011; Kuo et al., 2012). Under catabolic conditions, REDD1 promotes the assembly of the TSC1/2 complex, inhibiting mTOR and protein synthesis (Wang et al., 2006). Likewise, sestrin1 is also a glucocorticoid target gene that serves to inhibit mTOR signaling by increasing the activity of the TSC1/2 complex (Budanov and Karin, 2008; Braun et al., 2011; Kuo et al., 2012). Sestrin1 activates AMP-responsive protein kinase (AMPK), which phosphorylates Tsc2, increasing its GAP activity and thereby suppressing mTOR. In addition to its catabolic function, KLF15 also possesses anti-anabolic activity, which it achieves via regulation of the mammalian target of rapamycin (mTOR) complex (Shimizu et al., 2011). Branched chain amino transferase 2 (BCAT2) is a mitochondrial enzyme that catalyzes the initial step in branched chain amino acid (BCAA) degradation and is transcriptionally regulated by KLF15. BCAAs stimulate the activity of mTOR, increasing translational initiation. The fall in BCAA levels in response to glucocorticoid signaling inhibits mTOR, subsequently decreasing protein synthesis. Indeed, supplementation with BCAAs improves mTOR activation and attenuates muscle atrophy in response to treatment with glucocorticoids (Shimizu et al., 2011). The regulation of protein synthesis by mTOR does not occur in isolation, as supplementation of BCAAs not only suppresses dexamethasone-induced mTOR inhibition, but also prevents the induction of MAFbx and MuRF1. Therefore, it is clear that glucocorticoids exert a significant component of their catabolic effect via inhibition of mTOR signaling. However, significant cross-talk between protein degradation and synthesis occur such that the two are coupled to avoid futile cycling of amino acids.

### Generation of oxidative stress

The generation of reactive oxygen species within catabolic muscle is also a significant driver of atrophy in response to inflammation or disuse (Li et al., 2005; Suzuki et al., 2007) and antioxidant administration attenuates muscle atrophy in response to LPS administration (Jin and Li, 2007). Oxidative stress produces atrophy principally via activation of p38 mitogen activated protein kinase (MAPK), which subsequently induces the expression of MAFbx, MuRF1 as well as the autophagy-lysosome system (Li et al., 2005; McClung et al., 2010; Doyle et al., 2011). While few studies exist demonstrating that glucocorticoids induce oxidative stress in skeletal muscle, there is an extensive literature documenting this process in other tissues (You et al., 2009; Almeida et al., 2011). It is therefore tempting to speculate that glucocorticoids may also induce atrophy via the generation of oxidative stress in muscle. This potential mechanism of glucocorticoid action on muscle represents and exciting avenue for future investigation.

### Myostatin

Myostatin is a small secreted protein produced in skeletal muscle that inhibits muscle growth (McPherron et al., 1997). Myostatin signals via the type II activin receptor, leading to the phosphorylation of the transcription factors SMAD2 and SMAD3 (Lee and McPherron, 2001; Rebbapragada et al., 2003). Through the action of these transcription factors, myostatin down regulates numerous genes involved in myogenic differentiation such as MyoD, myogenin and myf5 (Langley et al., 2002; McFarlane et al., 2006). Myostatin signaling is implicated as a crucial intermediary between glucocorticoid signaling and muscle atrophy. Putative GREs are present in the human myostatin promoter (Ma et al., 2001). Consistent with this, glucocorticoids upregulate myostatin expression, and increase myostatin promoter activity (Allen and Loh, 2011). Furthermore, myostatin knockout mice fail to exhibit any muscle atrophy in response to glucocorticoid administration (Gilson et al., 2007). Critically, it was recently demonstrated that treatment of cancer cachexia in mice by the administration of a soluble type II activin receptor, improves survival independent from cancer progression (Zhou et al., 2010).

### Regulation of insulin signaling

It has long been appreciated that insulin and glucocorticoid signaling exert reciprocal forces on skeletal muscle mass. It has recently become clear however that rather than exerting this control via opposing, independent mechanisms, glucocorticoids fundamentally alter the transduction of signals downstream from the insulin receptor. Upon binding to their cognate receptors, IGF1 and insulin stimulate the recruitment of the insulin-receptor substrate -1 (IRS-1). After recruitment, IRS-1 is phosphorylated by the kinase activity of the insulin receptor, and serves as a docking site for PI3K. A recent genome wide analysis of GR binding identified p85α as a direct GR target gene (Kuo et al., 2012). Encoding the regulatory subunit of phosphatidylinositol 3- kinase (PI3K), p85α modulates insulin signaling via the relative abundance of inhibitory homodimers and active PI3K formed from p85α/p110 heterodimers. As such, overexpression of p85α under the influence of glucocorticoids, tips this balance in favor PI3K inhibition, resulting in decreased activation of the downstream kinase Akt. Of the many targets of Akt, perhaps the most important with regard to muscle atrophy are the Foxo transcription factors, which are excluded from the nucleus when phosphorylated by Akt, preventing them from transactivating the muscle-specific E3 ligases (Sandri et al., 2004). The glucocorticoid receptor also exerts DNA-binding independent effects on insulin signaling via direct interaction with PI3K (Hu et al., 2009). The effect of this interaction is to sequester PI3K subunits away from upstream signaling proteins in the insulin-receptor signaling cascade, preventing PI3K activation, leading eventually to Foxo1 nuclear localization.

### MicroRNAs as mediators of glucocorticoid-induced muscle atrophy

MicroRNAs (miRNAs) are small ~22 nucleotide RNAs that suppress mRNA expression via translational suppression and mRNA decay. Over the last decade, miRNAs have been shown to play a role in a diverse array of biological processes. Muscle is no exception to this, as recent reports have implicated miRNAs in the process of atrophy. In response to glucocorticoid treatment, miR-1, miR-147, miR-322, miR-351, and miR-503, miR-708 are found at increased levels in muscle cells (Shen et al., 2013). While the precise roles of many of these induced miRNAs has not been elucidated, the effect of miR-1 has been well characterized. One of the principle targets of miR-1 muscle is heat shock protein 70 (HSP70), which decreases in expression in response to glucocorticoid treatment (Kukreti et al., 2013). HSP70 binds to phosphorylated Akt, protecting it from dephosphorylation, and maintaining Foxo transcription factors in their phosphorylated, inactive state. The miR-1 mediated loss of HSP70 expression in response to glucocorticoid administration results in the inactivation of Akt signaling with subsequent nuclear localization of Foxo1 and induction of MAFbx and MuRF1. Other miRNA species appear to play a protective role against atrophy. miR-23a directly interacts with MAFbx and MuRF1 mRNA, inhibiting their expression and therefore the induction of muscle atrophy (Wada et al., 2011). Likewise, miR-27a and b also serve to inhibit muscle atrophy. Both of these miRNA species interact with the 3′ UTR of myostatin mRNA, decreasing its stability and half-life (Allen and Loh, 2011). In response to dexamethasone treatment, the levels of both miR27a and b decrease in skeletal muscle, facilitating an increase in myostatin expression, contributing to muscle atrophy. The role of miRNAs as mediators of muscle atrophy is a relatively nascent field with a great deal of that is as-of-yet unkown. However, early results suggest that they serve important roles as positive and negative regulators of muscle atrophy both via interaction with signaling pathways and by direct inhibition of catabolic mediators. Further dissection of the role of miRNAs in muscle atrophy will likely superimpose a new layer of complexity onto the transcriptional and translational regulation of glucocorticoid-induced muscle atrophy.

### Glucocorticoid regulation of muscle satellite cells

Increasingly, it is becoming appreciated that satellite cells, the resident stem cell population within skeletal muscle, play a critical role in the pathogenesis of muscle atrophy. In response to cancer growth, satellite cells are activated and enter the cell cycle (He et al., 2013). However, their fusion with the mature myofiber is impaired due to upregulation of the transcription factor Pax7. This appears to be dependent upon a circulating factor that is small and not susceptible to heat-inactivation. A second recent report demonstrates that synthetic glucocorticoids are potent inhibitors of satellite cell function (Dong et al., 2013). As glucocorticoids have recently been implicated in the pathogenesis of cancer cachexia, this provides a testable mechanism for satellite cell dysfunction in cancer (Braun et al., 2013).

## Glucocorticoids in disease

### Inflammatory cytokines, endotoxemia and sepsis

With the development of techniques for the production of recombinant cytokines, there was extensive interest in understanding the mechanisms by which they elicit muscle protein breakdown. These studies utilized an *in vivo* inflammatory challenge followed by the measurement of tyrosine release from an explanted muscle into culture media. Initial studies in which recombinant cytokines were given to animals in the presence of the GR antagonist mifepristone produced mixed results (Zamir et al., 1992). Administration of the glucocorticoid antagonist mifepristone attenuates the induction of muscle protein degradation brought about by tumor necrosis factor (TNF). In contrast, the proteolysis accompanied by interleukin-1 α administration was not blocked by concurrent mifepristone. However, the interpretation of these studies was complicated by mortality approaching 50% with concurrent cytokine and mifepristone administration, likely speaking to the necessity of glucocorticoid signaling for maintaining cardiovascular stability in the face of inflammatory challenge. When inflammatory cytokines are delivered directly into the brain, a rapid induction of MAFbx, MuRF1, and Foxo1 is observed in muscle and this induction is blocked by adrenalectomy or mifepristone administration (Braun et al., 2011). Additionally, the induction of these genes 8 h after peripheral lipopolysaccharide (LPS) administration is also blocked by adrenalectomy. However, other cytokine systems may produce muscle atrophy independent from the action of glucocorticoids. The TWEAK-Fn14 system potently induces muscle atrophy (Mittal et al., 2010). High doses of glucocorticoids fail to induce the TWEAK-Fn14 system. Further, it has yet to be investigated whether, like other inflammatory cytokines, glucocorticoid antagonism prevents TWEAK-induced muscle atrophy.

The role of glucocorticoids in muscle atrophy seen in experimental sepsis has also been investigated using a rodent cecal ligation and puncture (CLP) model. In juvenile rats, mifepristone significantly reduced tyrosine release from muscle explants harvested 16 h after CLP, arguing for glucocorticoids as a fundamental driver of this process (Tiao et al., 1996). Subsequent work demonstrated that the E3 ligases MAFbx and MuRF1 are also regulated by glucocorticoids in the juvenile CLP model (Wray et al., 2003). Contrasting results emerged from a CLP model in adult rats, where mifepristone failed to alter the induction of MAFbx and MuRF1 in septic rats despite effectively blocking the induction of these genes by dexamethasone at a similar time point (Frost et al., 2007). Although an explanation for these disparate results is not immediately apparent, it may reside in the relatively short 1–2 h half-life of mifepristone rodents (Heikinheimo et al., 1994). These studies examined gene expression at different time points (16 h after CLP in juvenile rats vs. 24 h in adults), a period of 4–8 half lives. It is further possible that sepsis significantly alters this half-life, rendering the drug effective at 16 h and not at 24 h. Continued work in the juvenile CLP model has demonstrated a significant induction of Foxo1 expression with PPARβ/δ playing a critical upstream role (Castillero et al., 2013).

A major limitation to this line of investigation has been the relatively blunt tools available for blocking glucocorticoid activity. Mifepristone has activity at other steroid receptors, and its use is limited by a very short half-life. Adrenalectomy can be used effectively at short time points after the induction of inflammation, however these animals are 1000-fold more susceptible to the lethal effects of inflammatory challenges, precluding the investigation of longer time points (Masferrer et al., 1992). Therefore, studies using mouse genetics to specifically interrogate the role of glucocorticoid signaling in skeletal muscle have clarified this question (Braun et al., 2013). Mice harboring a muscle-specific deletion of the GR display a significantly attenuated induction of MAFbx and MuRF1 in response to LPS administration. Further, given that glucocorticoid signaling is intact in other tissues in these animals, longer time points could be assessed after inflammatory challenge. In these experiments, the deletion of the GR in skeletal muscle largely prevents muscle mass loss or the loss of fiber cross sectional area in response to LPS administration. In total these results demonstrate the integral role for glucocorticoid signaling in muscle atrophy in the setting of acute inflammation.

### Diabetes

Acute diabetes arising from insulin deficiency has long been appreciated to produce glucocorticoid-dependent muscle atrophy. Adrenalectomized mice resist muscle atrophy in response to streptozotocin-induced diabetes (Hu et al., 2009). However, when the synthetic glucocorticoid dexamethasone is given in isolation at a dose estimated to mimic endogenous concentrations in diabetic animals, it is insufficient to evoke substantial atrophy. However, this same dose of dexamethasone restores normal muscle atrophy when administered to diabetic adrenalectomized mice. Through a series of elegant studies, it was demonstrated that the combined lack of anabolic insulin signaling and glucocorticoid signaling are necessary to produce atrophy in response to diabetes.

### Renal failure and metabolic acidosis

One of the hallmarks of chronic kidney disease (CKD) is a metabolic acidosis due to impaired renal acid excretion. Metabolic acidosis, like diabetes, was among the early conditions in which the elevated rate of muscle protein degradation was attributed to glucocorticoids. In these studies, animals subjected to experimental chronic acidosis demonstrated a significant increase in the rates of skeletal muscle protein degradation, which was prevented by adrenalectomy (May et al., 1986). Increased angiotensin II levels are also found in CKD, and implicated in the pathogenesis of muscle wasting in this condition. When angiotensin II is infused at concentrations mimicking endogenous production in CKD, significant muscle wasting occurs that is reversed by co-administration of the glucocorticoid antagonist mifepristone (Song et al., 2005). As of yet, there have been no studies directly assessing the contribution of glucocorticoids to muscle wasting in an experimental model of chronic kidney disease. However, in such models, urinary glucocorticoid excretion is elevated (May et al., 1987), with serum levels likely elevated into a range known to produce wasting in isolation. Therefore, with the advent of genetic models to investigate the impact of glucocorticoid signaling on skeletal muscle, such results are likely forthcoming.

### Cancer

Tumor growth is associated with a significant increase in systemic inflammation and elevated levels of circulating glucocorticoids. Therefore, it was surprising when initial studies using glucocorticoid antagonists or adrenalectomy failed to improve muscle mass in tumor bearing animals (Tessitore et al., 1994; Llovera et al., 1996; Rivadeneira et al., 1999). As a result, it was concluded that endogenous glucocorticoids do not contribute to muscle wasting in cancer, and a mechanism involving the direct action of cytokines on skeletal muscle came into favor. Recent work with muscle-specific GR knockout mice has returned the glucocorticoid hypothesis of cancer cachexia to the forefront. When the GR is deleted from skeletal muscle, muscle wasting in response to tumor growth is blocked (Braun et al., 2013). The discrepancy between these findings likely arises from the systemic effects of glucocorticoid signaling that are integral to the response to stressful stimuli and blocked in the setting of adrenalectomy or systemic antagonist administration. In addition, mifepristone has a very short half life in rodents on the order of 1–2 h, and therefore may be more appropriate for the investigation of acute events rather than chronic effects taking place over the course of weeks (Heikinheimo et al., 1994). Alternately, it is possible that some tumor models produce a glucocorticoid-dependent muscle wasting, while others do not, as different models were utilized in these studies. Future studies will likely expand upon the dependence of cancer cachexia on glucocorticoids and explore the applicability of these findings to the human condition.

### Direction of future work

Given the nodal nature of glucocorticoids as a regulator of muscle mass in chronic disease, there are a number of exciting avenues of research for future investigation. Principal among these is the development of a more refined understanding of the precise mechanisms of glucocorticoid-induced atrophy. Given the rapidly expanding field of non-genomic effects of steroid hormone receptors, future work will likely explore the interplay between the genomic targets of the GR and its non-genomic interaction with crucial signaling pathways. A second and equally interesting area of investigation will be resolving the apparent paradox for the requirement of glucocorticoid signaling as well as inflammatory signaling pathways it is known to inhibit. It will be interesting to discover whether there is synergy between NFκB and glucocorticoid signaling with regard to skeletal muscle atrophy and the precise molecular mechanisms of this effect.

A long-standing goal in glucocorticoid research is the development of novel GR agonists that serve as effective anti-inflammatory agents but have a reduced side effect profile. Given the extensive use of glucocorticoids as anti-inflammatory agents, and the significant toxicity associated with their use, any agent with an improved therapeutic index would be of significant clinical use.

The blockade of the catabolic effects of endogenous glucocorticoids is perhaps a taller order. The pleotropic effects of glucocorticoids on nearly every organ makes glucocorticoid antagonism a therapeutic approach encumbered by significant potential side effects. The ideal agent would displace cortisol from the GR in muscle, preventing catabolism while simultaneously allowing normal signaling in other tissues.

An interesting feature of glucocorticoid-induced muscle atrophy is that transcriptional activation dominates over transcriptional repression. It has been proposed that the anti-inflammatory and metabolic effects of glucocorticoid signaling can be separated on the basis transrepression and transactivation respectively. This concept originated with the finding that point mutations in the GR dimerization loop prevented dimerization and subsequent transcriptional activation of canonical glucocorticoid response elements, while maintaining the ability to transrepress NFκB and AP-1 dependent reporter constructs (Reichardt et al., 2001). Mice with the targeted A458T point mutation (GR^dim^) in the dimerization loop are viable in contrast to the perinatal lethality observed in the GR knockout mouse (Reichardt et al., 1998). Initial studies in these mice were very promising, as dexamethasone administration continued to suppress PMA-induced skin inflammation but failed to alter the expression of genes regulating carbohydrate balance (Reichardt et al., 1998; Tuckermann et al., 1999). The initial elegance of this model in which negative and positive effects are neatly partitioned into transactivation and transrepression respectively has unfortunately not born out. A study documenting the transcriptional response to prednisolone in GR^dim^ mice revealed marked variability with a subset of genes demonstrating an exaggerated response relative to wild type mice (Frijters et al., 2010). When GR^dim^ mice are treated with dexamethasone, there is a significant attenuation of weight loss compared with wild type mice (Waddell et al., 2008). In concordance with this, the induction of MuRF1 and Foxo1 in skeletal muscle by dexamethasone is markedly attenuated. However, GR^dim^ exhibit an equivalent degree of muscle atrophy, arguing against the hypothesis that dimerization dependent-transactivation is the sole mechanism for muscle atrophy. The concept that the anti-inflammatory action of glucocorticoids is dependent on transrepression has also recently been challenged. A large component of the anti-inflammatory action of glucocorticoids occurs as the result of transcriptional activation of the gene dual specificity phosphatase 1 (Dusp1) rather than the transrepression of NFκB and AP-1 (Hammer et al., 2006; Vandevyver et al., 2012). Mice harboring a deletion of Dusp1 demonstrate increased sensitivity to the lethal effects of LPS administration as a result of excess inflammatory cytokine production.

The emergent complexity glucocorticoid signaling makes the rational development of GR ligands with the ability to block the catabolism of muscle difficult. It is clear that a model in which desirable and non-desirable effects can be dissociated on the basis of GR dimerization does not accurately reflect the biology of glucocorticoids. Despite this, many new ligands of the GR, termed selective GR modifiers (SGRMs) have been developed, each with its own unique gene activation and repression profile. While such compounds have yet to be characterized in skeletal muscle, there is reason to be hopeful that via the careful combination of *in vitro* and *in vivo* screening, anti-catabolic GR ligands will be identified. Given the growing evidence for the central role of glucocorticoids as mediators of muscle atrophy, and the clinical burden of wasting disorders, the need for further research on such agents is high.

## Author contributions

Theodore P. Braun and Daniel L. Marks wrote and edited the manuscript.

### Conflict of interest statement

The authors declare that the research was conducted in the absence of any commercial or financial relationships that could be construed as a potential conflict of interest.
